# Nurses’ practice of integration of HIV prevention and sexual and reproductive health services in Ntcheu District, Malawi

**DOI:** 10.4102/curationis.v42i1.1919

**Published:** 2019-07-18

**Authors:** Sinegugu E. Duma, Lapani C. Ngala

**Affiliations:** 1 School of Nursing & Public Health, College of Health Sciences, University of KwaZulu-Natal, Durban, South Africa; 2 Ntcheu District Hospital, Ntcheu, Malawi

**Keywords:** sexual and reproductive health services, HIV prevention, integration, nurses, resource-constrained countries

## Abstract

**Background:**

Nurses play a critical role in their practice of integrating human immunodeficiency virus (HIV) prevention and sexual and reproductive services to combat the spread of HIV and promote family planning in resource-constrained countries like Malawi.

**Objectives:**

The purpose of this study was to determine and describe the nurses’ practice of integration of HIV prevention and sexual and reproductive health (SRH) services as a strategy to effectively combat the spread of HIV and promote family planning in Malawi.

**Methods:**

A descriptive qualitative case study was used. The research question was: How do nurses practise the integration of family planning and HIV prevention services in Ntcheu District, Malawi? Qualitative data were collected using semi-structured interviews from a sample of 10 participants. Manual data analysis, using the five steps for interpretive content analysis, was used to analyse data.

**Results:**

Five themes were identified as (1) facilitation of access and acceptability of comprehensive HIV and family planning services, (2) educating and counselling patients, (3) early detection of HIV among women of child-bearing age, (4) professional benefits of integrating family planning and HIV prevention services and (5) resentment of integration of family planning and HIV prevention services.

**Conclusion:**

The nurses’ practice of integration of HIV prevention and SRH services has more benefits for both nurses and patients as a strategy to combat the spread of HIV and promote family planning in a resource-constrained country like Malawi.

## Introduction and background

The integrated human immunodeficiency virus (HIV) prevention and sexual and reproductive health (SRH) services deliver a coordinated response to the dual challenges of high HIV prevalence and unmet needs for family planning. It widens the reach of HIV prevention and family planning messages to all communities (Liambila et al. 2008:22). Integration involves combining HIV risk assessment, HIV testing and counselling, treatment and care, and SRH services like family planning, maternal and child health, and management of sexually transmitted infections (STIs) within the health sector to offer comprehensive health services at one service delivery point or through a strong referral system (International Planned Parenthood Federation [IPPF] 2014). Integrated services expand access to and coverage of critical services and improve the efficiency of such services by reducing duplication of service delivery functions while delivering more services per client contact (Lusti-Narasimhan, Colins & Hopkins 2014:88).

Nurses are in the forefront of service provision for HIV prevention and family planning in many African countries. Some of these countries, including Malawi, where this study was conducted, have adopted and implemented the integration of HIV prevention and SRH services because it is considered as a promising practice to address unmet needs for contraception and to reduce mother-to-child transmission of HIV (World Health Organization [WHO] 2009:4). Unfortunately, not much has been recorded on the nurses’ actual practice of integration of HIV prevention and SRH services in the healthcare human resource-constrained African countries, like Malawi.

A rapid assessment conducted by the Centre for Reproductive Health, in collaboration with the International Planned Parenthood Federation (IPPF), United Nations Population Fund Activities (UNFPA) in 2010 and 2011 in Malawi, to determine whether patients accessing HIV services were also able to access family planning services either on site or through referral mechanism identified gaps and areas of improvement needed in that country. These included improving coordination between Ministry of Health’s Reproductive Health Directory (RHD) and HIV and AIDS department, training providers to provide integrated services and using task shifting to expand access to services (Centre for Reproductive Health 2010; Mutemwa et al. 2013). Later, the 2015 policy review on family planning and HIV integration in Malawi identified various types of HIV services, such as counselling on HIV prevention, voluntary counselling and testing (VCT) and dispensing antiretrovirals (ARVs), to be integrated into existing family planning services (Iran, Pappa & Dindi 2015:6). The Reproductive Health Service Delivery Guidelines of Malawi supports the need for provider-initiated counselling and testing among family planning patients, who do not know their HIV status. The guidelines highlight that family planning providers, including nurses, should be trained in counselling and testing for HIV, and that there should be proper referral of patients to pre-antiretroviral therapy or antiretroviral therapy (pre-ART/ART) clinics, while HIV-negative women should be counselled on risk reduction and dual protection. The guidelines also state that a clear referral mechanism should be established when certain HIV services cannot be provided at the same site as family planning in that country (Iran et al. 2015:6).

In response to the Malawi National Policy on Family Planning and the Malawi HIV integration and Sexual and Reproductive Health Service Delivery Guidelines, it became necessary to determine and describe the nurses’ practice of the integration of HIV prevention and SRH services as a strategy to effectively combat the spread of HIV and promote family planning in that country.

## Research methods

A descriptive qualitative case study was conducted in one family planning clinic within the secondary level hospital in the district health office of Ntcheu, Malawi. The research question was: How do nurses practise the integration of family planning and HIV prevention services in Ntcheu District, Malawi?

A modified conceptual framework for assessing facilitators and barriers to knowledge use by Cabana et al. (1999:1458–1465) was used to guide the study. A descriptive qualitative case study design was found to be the most suitable design as it has already been used in organisational studies (Yin 2009:17). The design allowed the researchers to gain an in-depth understanding of the nurses’ practice of integration of HIV prevention and SRH services as a ‘contemporary phenomenon’ within a ‘real-life context’ of the selected secondary hospital in Ntcheu District, Malawi (Yin 2009:18). The single unit of study was the nurses’ provision of integrated family planning and HIV prevention services, and the context was the practice of family planning and HIV prevention care. The family planning clinic within the secondary hospital and timeframe for data collection formed the boundaries of the case study. The features of the case that were examined were knowledge of and attitudes and perceived barriers to practising integration of family planning and HIV prevention services as three domains of the modified conceptual framework of Cabana et al. (1999:1458–1465).

### Population and sampling

The targeted population were all nurses providing SRH services and HIV services at the Ntcheu District Hospital during the time of data collection from February to May 2016. Purposive sampling, based on the study objectives and the research question, was used to select and recruit the participants who met the criteria to be included in the study and could provide rich information required to answer the research question.

As defined by Guest, Bounce and Johnson (2006:59), a sample of 10 participants, including the two participants from the pilot, was reached through attaining data saturation. This small sample size is accepted in qualitative research and facilitates close relationship between the researcher and the participants, thus enhancing the validity of the findings (Mackenzie & Crouch 2006:484).

### Data collection tool

A semi-structured interview guide was developed by the researchers in line with the research objectives and the concepts of the modified conceptual framework for assessing facilitators and barriers to knowledge use by Cabana et al. (1999:1458–1465) that was used to guide the study.

The pilot study was conducted in the last week of February and first week of March 2016, using two participants who were purposively selected and met the inclusion criteria of the study. As recommended by Van Teijlingen and Hundley (2010:102), the pilot study was conducted to allow for the refinement of the semi-structured interview guide if necessary. However, data analysis of pilot data showed that it was not necessary to refine the semi-structured interview guide. This was confirmed by the senior author. The data from the pilot study were added to the data of the main study because the process of data collection was the same in both the pilot and main study.

### Data collection and data management

Data collection and preliminary data analysis were conducted between March and May 2016 when the participants were off duty. Individual interviews were conducted with all participants individually to get first-hand information. Observations of non-verbal communication during the interviews were noted as field notes. English was used to conduct interviews because it is the only official language used in the healthcare system in Malawi. Interviews were audio-recorded and transcribed within 24 h, while the information was still fresh in the mind of the researcher. Each transcribed interview was sent to the senior researcher immediately for guidance in determining how collected data were in line with the research objectives. Preliminary data analysis was conducted parallel to data collection in the same manner as described in the ‘Data analysis’ section. This was to enhance sampling until data saturation was attained.

### Data analysis

Formal data analysis commenced soon after completion of data collection and preliminary data analysis which was conducted during the process of sampling and data collection to determine data saturation. Manual data analysis (except for the use of Microsoft Word to store and retrieve coded data), using the five steps for interpretive content analysis as outlined by Terre Blanche, Durrheim and Painter (2006:322–325), was used to analyse the transcribed data.

Firstly, the transcribed interviews and field notes were read many times to gain in-depth familiarity with the collected data. Notes on identified meanings were made reflectively where appropriate in the margins of the sheet containing the data. This was followed by coding which involved underlining phrases and sentences that were found to be relevant to the research question and objectives in the transcripts from all participants, starting from the first to the last transcript. Different colours were used to represent each identified statement. Each colour was given a label to ensure identification of which code belonged to which statement within each participant’s transcript. Numbering of the individual codes was done for purposes of storage and retrieval for within-case and across-case analysis, resulting in 42 identified codes. As proposed by Miles and Huberman (1994:64), a second qualified and experienced qualitative researcher was employed as an inter-coder. The codes’ applicability was tested on the two transcripts from the pilot study. The codes from both the researcher and the inter-coder were further verified and confirmed against raw data by the senior researcher who is also the second author.

The identified codes were grouped to generate categories based on their similarity in meaning. The categories were then grouped to form themes, based on similarity. The whole process was submitted to the senior researcher for verification. Individual themes were further interpreted for meaning to construct an exhaustive, in-depth understanding of the nurses’ practice of the integration of family planning and HIV prevention services using each objective of the study and the research questions to construct an inferred meaning of the phenomenon studied within the context of the secondary level hospital in Ntcheu District, Malawi.

This extensive data analysis process yielded five themes. Member checking, which refers to verification of findings by the participants (Holloway & Wheeler 2013:280), was conducted with the seven participants who were still available at the research setting. They all confirmed that the five themes reflected what they believed to be the nurses’ practice of integrating family planning and HIV services in the secondary level hospital in Ntcheu District, Malawi.

### Trustworthiness of the study

The four basic frameworks for ensuring rigour, which is quality, authenticity and truthfulness of findings, were adhered to throughout the study. This includes transferability, credibility, confirmability and dependability (Shenton 2004:73). Among other things, member checking was done by going back to participants with the findings to check and verify the interpretation of the researchers (Holloway & Wheeler 2013:280). It was found that the interpreted data reflected what the participants believe is the nurses’ practice of integrating HIV prevention and SRH services in the secondary level hospital in Ntcheu District, Malawi. Inter-coding agreement between the researcher and inter-coder was reached and further confirmed by a senior researcher. This ensured that the codes were a true reflection of data from participants. The complete set of analysed data was further checked, and the authenticity of identified themes was confirmed by the senior researcher who is also the senior and second author of this article.

### Ethical considerations

Ethical clearance (HREC REF: 873/2015) was obtained from the relevant authorities. All ethical principles for conducting research with human subjects as described in the Declaration of Helsinki of 2013 were observed throughout the study (World Medical Association 2013). Each participant signed and provided a written informed consent form to participate in the study.

## Results

Of the total of 10 participants, including the two participants from the pilot study, two were male and eight were female nurses who have worked in the family planning clinic, a research setting, for a period of between 2 and 8 years as qualified and licensed nurse practitioners. All participants reported having received in-service training on the integration of family planning and HIV services. The age of the participants ranged between 25 and 49 years.

Five themes were identified as (1) facilitation of access and acceptability of comprehensive HIV prevention and family planning services, (2) educating and counselling patients, (3) early detection of HIV among women of child-bearing age, (4) professional benefits of integrating family planning and HIV prevention services and (5) resentment of integration of family planning and HIV prevention services.

### Facilitation of access and acceptability of comprehensive HIV prevention and family planning services

This theme was derived from data related to nurses’ practice of facilitating the access and acceptability of comprehensive HIV prevention and family planning services as captured in the following extracts from the data:

‘We ensure that every woman of child-bearing age who comes to our family planning clinic has access to family planning services as well as HIV prevention services at any time and any day … we also ensure that these women have access to the family planning method they want at any time.’ (Participant B, male, aged 24 years old)

The following extract demonstrates nurses’ familiarity with facilitating access and accessibility of comprehensive integration of family planning and HIV prevention services by helping women to select the most appropriate family planning method.

‘But there are some family planning methods which not every woman was familiar with previously but they are now able to access even when they tested HIV negative. So we do family planning and HIV counselling daily to ensure that the HIV-negative patients are given the right choice of family planning method.’ (Participant D, female, aged 32 years old)

The theme was further supported by the following quote from data:

‘With the coming of integration of family planning and HIV prevention services, nowadays we ensure that all women who come for family planning services have access of both family planning and HIV counselling and testing services on a daily basis.’ (Participant F, female, aged 27 years old)

### Educating and counselling patients

This theme emerged from data that related activities carried out by nurses to educate and counsel patients as part of integration of HIV prevention and reproductive health services, such as giving motivational talks on the importance of getting tested for HIV, teaching patients about the advantages and disadvantages of all family planning methods, helping patients to choose the best family planning methods according to their HIV status and continuous counselling on positive living. The following extracts from the data highlighted the theme ‘Educating and counselling patients’ during integration of family planning services with HIV services:

‘We give a general health talk and later a motivation talk on the importance of getting tested for HIV and spacing your children well.’ (Participant A, female, aged 24 years old)

Educating and counselling patients about family planning and HIV prevention services was further captured through the following extract from data:

‘Each and everyone who comes for family planning is counselled and tested for HIV and given a motivation talk on the importance of getting tested for HIV. That’s what we do that all the time.’ (Participant E, female, aged 29 years old)

Another extract expressed similar sentiments as follows:

‘When these women come for the family planning clinic we give them a health talk on all the available family planning methods, their advantages and disadvantages and then later a motivational talk on the importance of getting tested for HIV, and this is our routine.’ (Participant G, male, aged 27 years old)

Another quote supporting the development of the theme ‘educating and counselling patients’ on integration of family planning and HIV prevention services was as follows:

‘At times we come across women who insist on using implants even when they have tested HIV positive and we have explained about the disadvantages of implants to HIV positive persons. We try to explain again and again until they can choose a method appropriate for them, we don’t just force them.’ (Participant F, female, aged 27 years old)

### Early detection of HIV among women of child-bearing age

This theme emerged from data that related to nurses’ activities related to early detection of HIV among women of child-bearing age, including retesting for HIV after every 3 months, screening for chronic conditions including HIV and knowing HIV status of all family planning patients, as shown in the following extracts from the data:

‘When someone tests negative for HIV, we congratulate them, but we tell them that even if they have been found negative, that does not mean they cannot get infected, but to continue preventing the virus but also getting retested after every three months. We insist on this because it may help with early detection of HIV among women of child-bearing age.’ (Participant A, female, aged 24 years old)

‘When a family planning client comes in, we weigh her, check blood pressure and test for HIV. She also gets counselled and tested for HIV before she receives her method of choice for family planning. In many instances, from doing this, we are able to detect other illnesses early.’ (Participant B, male, aged 24 years old)

‘Nowadays all patients who come for family planning undergo HIV counselling and testing before they receive a method of their choice. This helps with early detection of HIV for early treatment.’ (Participant D, female, aged 32 years old)

Early detection of HIV among women of child-bearing age women was further captured from the following extract:

‘With the coming of integration of family planning and HIV prevention services we are able to detect HIV among our patients at an early stage through screening when they are not yet sick, and this makes work easier.’ (Participant E, female, aged 29 years old)

### Professional benefits of integrating family planning and HIV prevention services

This theme was derived from data that related to professional benefits of nurses’ practice of the integration of family planning and HIV prevention services, as captured in the following extracts from the data:

‘If you identify positive woman who comes for family planning, and as a provider you discover that she is also breastfeeding, but was missed for HIV counselling and testing during antenatal care, with this approach, you are able to assist her accordingly, without having to refer her to someone else.’ (Participant A, female, aged 24 years old)

Another extract from data highlighted the benefit of picking up on missed patients as a benefit of family planning and HIV service integration, as shown in the following extracts from data:

‘For example let’s say a mother was missed on HIV counselling and testing during antenatal care and comes to family planning clinic with her breastfeeding baby, gets tested and is found HIV positive … now with integration it means this mother will be put on ART and baby on Bactrim.’ (Participant B, male, aged 24 years old)

The following quotes were extracts that were also identified as benefits of integrating family planning and HIV services by nurses in Malawi:

‘Women who are missed during antenatal care or were on window period, we are able to capture them during family planning clinics … this protects their babies as well as the whole family because they will have a healthy life now.’ (Participant C, female, aged 30 years old)

‘Those mothers missed during antenatal are being captured now with this integration thereby preventing mother to child transmission.’ (Participant E, female, aged 29 years old)

The following extracts highlighted the reduction of HIV-related deaths as a benefit of integration of family planning and HIV services by nurses in Malawi as follows:

‘With integration of family planning and HIV prevention we are preventing or reducing HIV-related deaths because patients are being identified at an early stage. That makes me happy knowing that through my practice, lives have been saved.’ (Participant A, female, aged 24 years old)

‘Integration of family planning and HIV services is a good thing because we are able to identify HIV-positive patients at an early stage when they are not sick, and this will reduce HIV-related deaths in our community.’ (Participant C, female, aged 30 years old)

‘If these women are accessing both family planning and HIV prevention services, this motivates me to do more for my people.’ (Participant H, female, aged 26 years old)

Another professional benefit of integration, that of reduction of workload, was captured in the following extracts from the data:

‘But there is also reduced workload. Previously without integration of family planning and HIV prevention of services we were having family planning only three times a week, and during these family planning days you could provide services to 50 patients. But now with integration the services are being provided on daily basis; we only see about 15 patients or less than that a day, so to me this is motivating. That is good for all of us.’ (Participant B, male, aged 24 years old)

‘Integration of family planning and HIV prevention of services seems to reduce workload at times because we are providing all the services on a daily basis … patients come in small groups than before integration, where you could have a big group of patients on a day since family planning clinics were being conducted twice a week.’ (Participant J, female, aged 23 years old)

### Resentment of integration of family planning and HIV prevention services

This theme was derived from data that related to the participants’ resentment or hatred of integration of family planning and HIV prevention services, which are captured in the following extracts from the data:

‘As for me, I see integration as a very good thing even though it seems like it’s too involving and needs more time to be spent with one patient. In that regard I think it is not as good as it was made to be when introduced to us.’ (Participant C, female, aged 30 years old)

‘Integration of family planning and HIV prevention is a good thing because our patients are receiving more than one service at one visit – only that it requires more time and it’s too involving at times. You end up having spent all your time and energy with one client.’ (Participant E, female, aged 29 years old)

‘As much as our patients are killing two birds with one stone, integration is exhausting to me as a provider. It drains all my energy due to increased workload… this makes integration of these services a pain to me, I don’t know about others.’ (Participant F, female, aged 27 years old)

‘Yes integration of family planning and HIV prevention is a good thing as it is improving the programme indicators, but to tell the truth, it leaves us nurses very exhausted with increased work.’ (Participant H, female, aged 26 years old)

## Discussion

The findings revealed that nurses’ practice of integration of family planning and HIV prevention services enabled them to facilitate access to and acceptability of HIV prevention and SRH services among patients. The practice was enhanced by the training in both family planning and HIV management, which the participants had received. Similar findings have been reported in many studies globally, where the integration of family planning and HIV prevention services has been acknowledged as a strategy to increase access to and acceptability of comprehensive integration of family planning and HIV prevention services for patients (Fullerton, Fort & Johal 2003:148; Kinagwi & Kibet 2011:58). The negative effects of limited or no training of nurses on integration of HIV prevention and SRH services were reported in some African countries, including Tanzania, Ethiopia, Kenya, Rwanda and South Africa (Holt, Lince & Hargey 2011; Kennedy et al. 2011; Nielsen-Bobbit, Kikumbih & Motta 2011). Clearly, training of nurses on the integration of HIV management and family planning are key to improving the nurses’ practice of integration of HIV prevention and SRH services and facilitation of access to comprehensive integrated HIV prevention and SRH. The WHO recommends that health providers should facilitate access to HIV counselling and testing to all women of child-bearing age. This not only facilitates access to HIV counselling and testing for all women of child-bearing age, but also maximises the health and well-being of individuals through the timely detection of HIV, prevention of HIV transmission and subsequent access to appropriate HIV prevention and treatment (WHO 2007).

The UNPFA’s (2013) recommendations support the nurses’ practice of educating and counselling patients of family planning and HIV prevention services as an important aspect in the integration of HIV prevention and SRH services. It further recommendations that health providers must share complete information regarding contraceptive method effectiveness as well as the advantages and disadvantages of each method with their patients (UNPFA 2013:4).

The importance of the findings on nurses’ practice of early detection of HIV is further highlighted by other authors. For instance, Ramfolo et al. (2011:3) suggest that knowing the patients’ HIV status has benefits for both the client concerned and the provider. For HIV-negative people knowing their status empowers them to protect themselves from becoming infected and to remain negative. For HIV-positive people knowing their status ensures that they can be provided with appropriate treatment, care and support services and be assisted in living positively (Ramfolo et al. 2011:3).

The reported professional benefits of implementing the integration of HIV prevention and SRH services, including patients coming in small manageable numbers, were reported to help nurses to feel good about helping their patients and to increase the trust between nurses and their patients, and are well supported in the literature. For instance, Nielsen-Bobbit et al. (2011) reported that a reduced number of multiple return visits or referrals were also reported in Tanzania, where health providers noted that integration of family planning and HIV services resulted in an increase in client uptake of services and reduced need for multiple return visits or referrals.

Similar findings on benefits of integrating family planning and HIV prevention services were reported in a longitudinal study in Haiti, where Peck, Fitzgerald and Liataud (2003:89) found that integrating additional services including family planning within HIV prevention programmes increased the uptake of HIV testing by 62 times over a 15-year period. In Zimbabwe, it was reported that offering integrated services through community health workers contributed to increases in contraceptive use and referrals to VCT centres and led to improvements in client attitudes and knowledge about both family planning and HIV prevention (United States Agency for International Development [USAID] Extending Service Delivery Project 2011). In Kenya and the Kingdom of eSwatini, it was found that client satisfaction improved because of the increased efficiency of services, which, in turn, had a positive effect on health workers’ own satisfaction (Kuria 2011; Mengistu, Mengistu & Nouga 2011; Scholl & Cothran 2011).

This study also revealed resentment of integration of family planning and HIV prevention services among some nurses. For instance, it was reported that the practice of integration of family planning and HIV prevention is too involving and takes more time, thus increasing the workload and resulting in exhaustion. Resentment of integration of family planning into VCT for HIV as burdensome, tiresome and time-consuming has been reported by others (Abera & Mengistu 2006; Nielsen-Bobbit et al. 2011).

## Limitations of the study

Only eight participants were reached for member checking. The two other participants were on vacation. Waiting for their return from vacation would have delayed the conclusion and reporting of the findings. It is common in member checking with participants who work in one institution and have to take vacation at different times.

## Recommendations

The nurses’ practice of the integration of HIV prevention and SRH should be promoted as a strategy to combat the spread of HIV and promote family planning in other resource-constrained countries.

## Conclusion

This study revealed the important aspects of nurses’ practice in the integration of HIV prevention and SRH services to combat the spread of HIV and promote family planning in Malawi.
